# Entomological Contributions to the Legal System in Southeastern Spain

**DOI:** 10.3390/insects12050429

**Published:** 2021-05-10

**Authors:** María-Isabel Arnaldos, María-Dolores García

**Affiliations:** 1 Area of Zoology, Faculty of Biology, University of Murcia, 30100 Murcia, Spain; mdgarcia@um.es; 2 Unit of Forensic Entomology and Evidence Microscopic Analysis, External Service of Forensic Sciences and Techniques, University of Murcia, 30100 Murcia, Spain

**Keywords:** Forensic Entomology, Spain, experimental studies, cases

## Abstract

**Simple Summary:**

Insects and other arthropods found at a forensic scene are considered to represent relevant evidence regarding the time and place of death, a possible antemortem or postmortem treatment of the victim, the geographical origin of narcotic substances, drugs, etc. However, in order to derive firm conclusions from entomological evidence, it is critical to understand the different aspects of the insect biology of the whole area under consideration. Moreover, most forensic investigators are not specially trained in basic entomology procedures, which can result in limitations when trying to achieve an accurate expert report. The present work illustrates the utility and necessity of such entomological studies and the expert training that is required using actual forensic cases.

**Abstract:**

The aim of this work is to present a number of forensic cases that took place in Southeastern Spain (Murcia province) in which the entomological evidence aided to fully solve the issues raised during the legal enquiry, enhancing the close interrelationships between experimental studies performed and actual forensic cases assessed. In all cases, the expert report was requested by the police agents or the medical examiners, the latter attempting to make stronger their own conclusions. The assessment of all cases was possible by comparing the evidence and circumstances of each one with the experimental data previously obtained in our laboratory concerning aspects such as faunistic, ecological, morphological, etc., and by considering data from other researchers. In all cases, the evidence could be addressed, although in some cases, it had not been properly collected or processed. Thus, the utility of the experimental studies in forensic practice, even when being considered merely biological, and without immediate practical application, can be demonstrated as well as the need for providing specialized instruction on Forensic Entomology procedures to the different agents involved in forensic investigation.

## 1. Introduction

Although the application of entomology to forensic practice has been largely referred to in the scientific literature in many countries since the 1970s [1], the legal system in Spain considers the study of entomological evidence as a supplementary technique. As a matter of fact, entomological evidence is rarely collected at the crime scene or during the autopsy procedure. For this reason, an important source of data may be lost during the enquiry, making it more difficult, or even impossible, to get an accurate diagnosis of the actual case under investigation. Until now, the scope of recognition and the inclusion of Forensic Entomology practices into the routine of a forensic investigation has been limited in Spain. Nevertheless, there are medical examiners and members of the State security forces who are willing to implement a standardized recovery of entomological evidence to aid in those forensic cases in which they participate. Sometimes the Forensic Entomology teams are required to study some entomological evidence from actual forensic cases; as an example, the medical examiner may be interested in complementary entomological evidence to reinforce their own conclusions and make their own results and conclusions of the entomologist’s expertise [1]. In such cases, the problem can arise when the evidence has not been properly recovered or processed or does not represent the whole entomological fauna related to the corpse. This could occur as a consequence of the lack of specific training on the subject from those recovering the samples, as reported, e.g., in García et al. [1].

Regardless of the source of evidence, the role of forensic entomologists is to study it and provide an independent expert’s opinion, most often related to the estimation of the postmortem interval (PMI). This estimation can be made on the basis of the age of an immature insect fed on the corpse, which provides a minimum PMI, and the carrion arthropod succession model, which could suggest a maximum PMI [2]. Succession data have been used to very accurately calculate a PMI as large as 52 days [3] and could be applied to a much larger interval [2] if the seasonal dynamics and succession of the entomosarcosaprophagous fauna in a given area are accurately known. In order to apply any of the two methods, previous information and knowledge are needed on larval growth rates, and how they are influenced by temperature, as well as on carrion succession. Then, previously existing experimental data are usually searched [2]. It needs to be considered that a PMI does not always correspond to the period of insect activity (PIA) [4] because the time when insects first colonized a body may not necessarily happen at the time of death. It could occur within minutes of being dead or could be delayed under certain circumstances (e.g., body buried or wrapped or concealed indoors) [4]. The PIA can eventually agree with the minimum PMI. As regards the developmental pattern of a certain species, there is abundant information for different taxa. Nevertheless, further efforts are needed in order to cover as many different species as possible as well as to properly understand if the developmental pattern is affected by regional or geographical variations. In fact, and according to Grassberger and Reiter [5], it seems that intrinsic factors, such as geographic adaptation, could explain a difference in temperature-dependent development.

With regard to the carrion arthropod succession, much is known on the importance of the geographical region or biogeoclimatic zone, and even the particular habitat concerning a concrete case, on insect colonization of such carrion (i.e., [6,7,8,9]). It has been proposed that different environments or habitats can influence the patterns of community assemblage [10,11], illustrating the importance of performing studies on carrion entomofauna in a variety of habitats and scenarios in all regions in which Forensic Entomology is used [6].

In the Iberian Peninsula, there are reports of the contributions to the knowledge of the structure and dynamics of the entomosarcosaprophagous fauna in several areas (i.e., [7,9,11,12,13,14,15,16,17,18,19,20,21,22,23,24,25,26]), and, in at least one case [14], a relationship between experimental study and forensic cases’ resolution has been established. Moreover, studies on certain groups of insects have addressed the morphology of immature stages [27,28,29,30] and the development of certain species under controlled conditions (i.e., [31,32] among others). Finally, some case reports have also contributed to promoting the use of Forensic Entomology in Spain [1,33,34]. Despite all of the above, the use of entomological evidence for solving forensic cases in Spain is still almost a rarity due, at least in part, to the lack of proper taxonomic revisions of many groups of arthropods [35]. For this reason, disseminating cases resolved with the help of this entomological discipline is particularly relevant in the country.

The aim of this work is to present a number of forensic cases that occurred in Southeastern Spain (Murcia province) in which entomological evidence was involved to fully elucidate the issues raised during the legal enquiry. It is important to highlight the close interrelationship between the experimental studies on entomosarcosaprophagous fauna that have been previously carried out and the actual forensic cases to be solved. This relationship is demonstrated by the fact that the assessment of all cases was possible by comparing the evidence and circumstances of each one with the experimental data that had previously been obtained and reported.

## 2. Cases Presentation

The forensic cases presented below are grouped based on the type of previous studies that were found to have utility for their resolution.

### 2.1. Morphological and Faunistic Studies

Knowledge of the morphology of the different species is critical when dealing with actual forensic cases. In this respect, classical faunistic studies on sarcosaprophagous fauna provide excellent training for identifying such fauna. The following cases fit well in this section.

**Figure 1:**
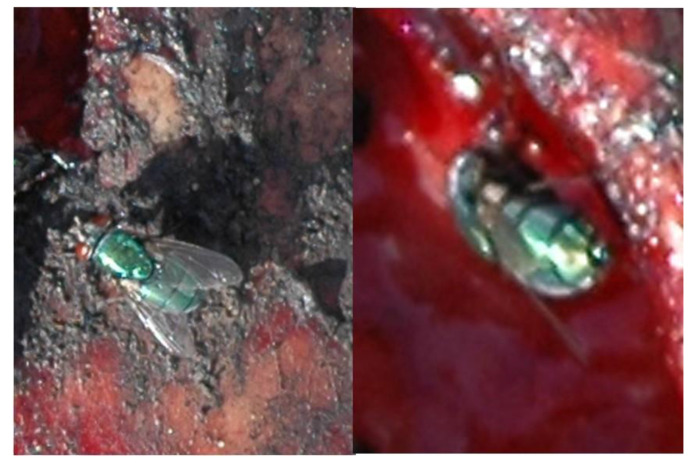
Photographs from graphic report by police agents.

- Case 1. A partly burned woman corpse was found in the early morning hours on the side of a road in the province of Murcia at the end of March. The police agents did not recover any entomological evidence but made an excellent graphic report from which different fly images could be studied. From these images (Figure 1)., and once magnified, *Chrysomya albiceps* (Wiedemann, 1819) was identified on the basis of our own experience and the dark hind stripe of the abdominal terga [36].
  Former faunistic studies [12,13] indicated that this species is the most common in the area, and efforts were directed to solving the case on the basis of this finding. None of the pictures revealed anything consistent with signs of activity of insects, such as eggs or larvae. According to what could be observed, the remains appeared to represent a fresh stage (despite having been superficially burned), suggesting a recent death. Because previous knowledge of fauna related to corpses in the area had pointed to a secondary character of *Ch. albiceps*, we consider the possibility that there could have also been some other, not graphically illustrated, activity of primary species. Nevertheless, and in agreement with Avila and Goff [37] and Pai et al. [38], who indicated that *Chrysomya* sp. can act as primary fly in burnt corpses, it could be concluded that the remains had been exposed for a short period. This conclusion was consistent with eyewitness testimony of a bonfire taking place in the location the same morning. The subsequent enquiry confirmed this conclusion.

- Case 2. García et al. [1] report the case of an unidentified mummified corpse found in a rural area of difficult access in Murcia province at the end of August. The police agents recovered some entomological evidence in situ from the corpse itself and below it, but this evidence was inadequate, and, as a result, two entomologists had to recover additional evidence from the corpse at the place where it was guarded. Among this evidence, a number of small fly pupae and puparia were studied. They could be identified as belonging to *Piophila megastigmata* McAlpine, 1978 on the basis of the already-existing morphological study of preimaginal stages of this species that allowed the identification of the found pupae thanks to the very wrinkled tegument (25–30 wrinkles, or even more, per abdominal segment) [29]. This finding represented the first report of such species on a corpse in Spain, and its presence was considered to estimate the PMI. For this purpose, a faunistic study of Diptera performed in the geographical area [39] was considered. This study stated that *P. megastigmata* could be present on corpses since the beginning of May. Given that the main question in the inquiry was whether the person had died on dates close to disappearance or at a later time, our findings were consistent with death likely occurring by the date of disappearance (at the end of May). Further details on the recovered fauna and the estimation of PMI can be found in García et al. [1].

### 2.2. Faunistic Succession and Developmental Data

Patterns of arthropod succession are considered a good method to estimate the PMI and can also provide valuable clues to an investigation [40] together with the essential accurate identification of taxa and with developmental data of species breeding on the corpse. Because some variation can occur in the colonization patterns due to the environmental and seasonal differences, PMI estimation on the basis of the succession data requires previous studies performed throughout the year on local carrion fauna [6]. According to Anderson [6] and Kreitlow [40], one of the most important challenges of Forensic Entomology is to provide the means for an estimation of the PMI or PIA as accurately as possible. In Murcia province, some projects of this kind have been addressed [13,41,42,43] and some forensic cases related to the aspects above mentioned are reported.

**Figure 2:**
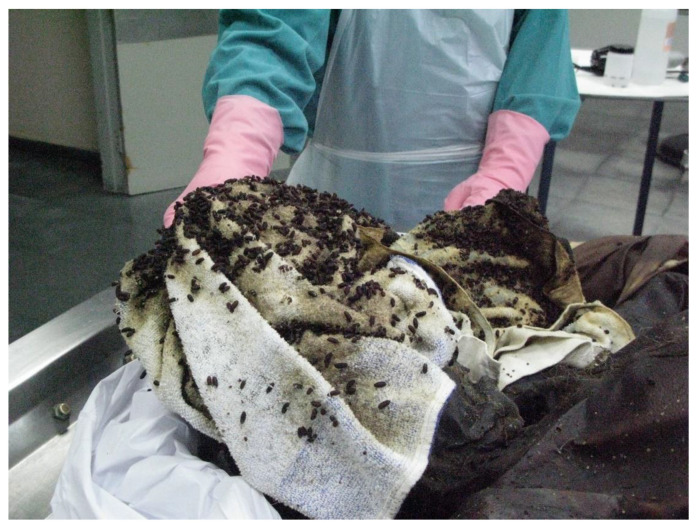
Autopsy procedure showing puparia from *Chrysomya albiceps*.

**Figure 3:**
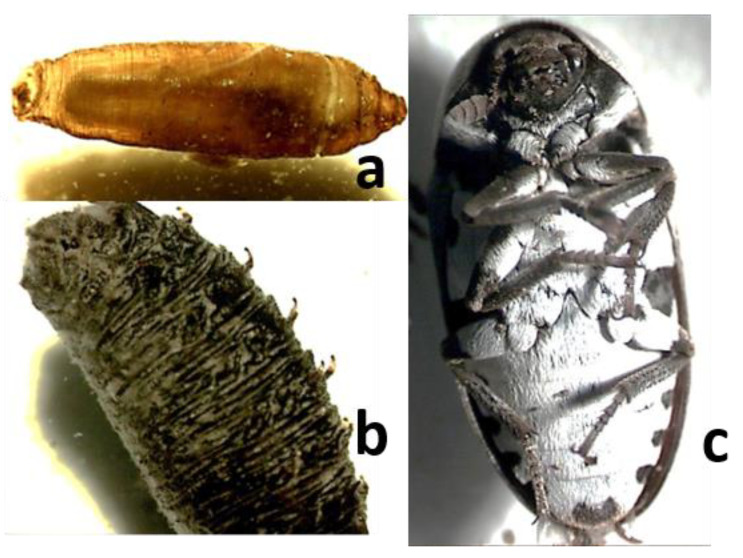
Entomological evidences (**a**) Piophilidae pupae (**b**) *Chrysomya albiceps* pupae (**c**) adult of *Dermestes frischi.*

- Case 3. This old case (2001) was initially referred to by Arnaldos et al. [14], but some of its results were later revised in light of new scientific findings (see below) that allowed properly referring to some taxa initially misidentified or simply not identified.
  The corpse of a homeless man was found in the middle of November at the bottom of a dry pot with some still water, next to Murcia City. The corpse was partially clothed and showed an incised wound in the abdomen. A recovery of entomological evidence was performed because two samples (one fixed and one alive) were provided from the autopsy procedure, although the larvae had been fixed in boiling ethanol. The fixed sample consisted of larvae of *Calliphora vicina* Robineau-Desvoidy, 1830 (LIII), *Lucilia sericata* (Meigen, 1826) (LII, II-III, and III), *Chrysomya albiceps* (LI-II and II), *Muscina stabulans* (Fallén, 1826) (LII, II-III, and III), *Synthesiomya nudiseta* (Wulp, 1883) (LII, II-III, and III) and *Sarcophaga argyrostoma* (Robineau-Desvoidy, 1830), Phoridae (adults, pupae, and LI and II),and an Acari unidentified. The living sample was kept in an incubator in the laboratory, where adults of several species (*Ch. albiceps*, *L. sericata*, *S. nudiseta*, and Phoridae) were obtained.
  Most larvae of the fixed sample were initially identified considering Smith [44,45], Reiter [46], and Greenberg [47]. At the moment of the expertise, *S. nudiseta* specimens were not identified at the specific level, and, in the case of Sarcophagidae larvae, accurate identification was impossible due to the lack of appropriate keys at that moment. The identifications of larvae were recently reexamined in light of findings from Velásquez et al. [48] mainly for *S. nudiseta*, Grzywacz et al. [49] for *Muscina* and Szpila et al. [50], and Ubero-Pascal et al. [28] for Sarcophagidae and confirmed the identification provided in the previous paragraph. Adults Calliphoridae were identified according to González-Mora and Peris [36] and Peris and González-Mora [51]. In the case of *S. nudiseta*, a first approach was made using keys from Gregor et al. (2002) [52] (they do not include that species) and later checked with the identified larvae and confirmed with Pont [53].
  In this forensic case, the recovered fauna fits with those characteristic of autumn in Murcia, formerly studied by Arnaldos et al. [13]. The corpse presented one open wound, which allowed the implication that the oviposition could have occurred immediately after death, in particular for *C. vicina* and *L. sericata*, primary species present on the corpses from the first day [12,13] in the region and that is known can coexist [54,55]. The species, as a whole, have been considered as belonging to the first waves of arthropods visiting a corpse [56]. Taking into account the oldest larvae of the primary species (*C. vicina* and *L. sericata*) and the mean temperatures registered in the area (at around 15 °C), and according to developmental data due to different authors, [57] among them, the PMI_m_ was estimated to be 11–17 days before the corpse was found. Despite the newly identified taxa, similar conclusions to previous ones were reached because these were based on *L. sericata* and *C. vicina* developmental data.
- Case 4. A male corpse was found at a home in Yecla (Murcia province) in the middle of September. The corpse was partly clothed and appeared to have been attacked by an animal, likely a dog. The man was last seen alive about 15 days earlier. Entomological evidence was recovered during the autopsy procedure and consisted of an adult female, three pupae, and several puparia of *Chrysomya albiceps*, one adult specimen of *Necrobia rufipes* (De Geer, 1875), and only five larvae, well developed, of *Dermestes* sp. (Figure 2).
  The best available evidence to estimate the PMI would be *Ch. albiceps* (for identification keys see above) that had clearly come to the end of its cycle. This species is the most common and abundant at the end of summer and during autumn [12]. Therefore, its presence in the corpse is congruent with the circumstances of the case. Taking into account the mean temperatures in Yecla during those days (20–25 °C) and developmental data of the species from several authors [58] and [59] among them, a PMI of around the time when the man was last seen alive was estimated. The fact of being inside a home was not considered relevant because the windows were open. Thus, it was reasonable to assume that the inside temperature was heavily influenced by the outside one.
- Case 5. A male corpse was found inside an abandoned old factory in Murcia province at the end of April. Entomological evidence was recovered during the autopsy procedure. As supplementary evidence, a complete graphical report was provided by the medical examiner in which abundant entomological specimens could be observed. The decedent was last seen alive about one month and a half earlier. The evidence consisted of four different samples, two of them directly fixed in ethanol (one from the clothes and the other from the body itself) and two being alive (one from the clothes and the other from the body). The fixed samples included *Synthesiomya nudiseta* (pupae), *Chrysomya albiceps* (LI, II, and III and pupae), *Piophila megastigmata* (pupae and adults), *Dermestes frischi* Kugel, 1892 (adults and larvae), and adults of *Necrobia rufipes* and Histeridae (*Saprinus* sp.). (Figure 3) Diptera species were identified as indicated above. Coleoptera species were identified according to Bajerlein et al. [60] and Hackston [61].
  From the living samples, kept at the lab, *Lucilia sericata*, *Chrysomya albiceps*, Phoridae, Chalcidoidea, and some unidentified Muscidae were obtained. The overall evidence was consistent with the sarcosaprophagous fauna characteristic of the season in the area [13] and suggested an advanced decomposition stage because Piophilidae, Dermestidae, and Histeridae, as well as aged preimaginal stages of Diptera, were present. Among all the taxa present, the most valuable for PMI estimation purposes were *Ch. albiceps,* of which some pupae were recovered. Adults emerged at the lab only two days after being kept in the incubator, suggesting that the pupae were very close to the end of the cycle. Taking into account the mean temperatures during the considered period (13.2 °C during March and 15 °C during April) and developmental available data (see former Case), a PMI_m_ consistent with the last time in which the decedent was seen alive was estimated.
- Case 6. A male corpse was found in an arid outdoor environment of Murcia province at the end of September. Entomological evidence recovered during the autopsy procedure was provided. Upon the request of the Forensic Entomology team, the medical examiner provided supplementary entomological evidence, proceeding of the moment of the corpse removal, and a graphical report, the latter providing an illustration of the dry decomposition stage of the corpse. The evidence from the autopsy consisted of two containers, one containing living specimens and the other individuals fixed in 80% ethanol. The evidence collected in situ consisted of one container with individuals that, although kept alive first, died at an unknown time and were preserved in 80% ethanol. The living sample was opened at the breeding lab, where it was found not to contain preimaginal stages of Diptera. This sample contained adults of *Necrobia rufipes* and Histeridae and adults and larvae of *Dermestes frischi.* The fixed sample from the autopsy contained adults of *Chrysomya albiceps*, *Cataglyphis* sp., and Histeridae and larvae of *Sarcophaga* sp. (LIII), *Lucilia sericata* (LIII), Piophilidae (LIII), and Dermestidae. The overall evidence is consistent with the dry stage of the corpse because Dermestidae larvae are characteristics of such stage, as well as *N. rufipes* and LIII of Piophilidae [62]). Aged larvae of Calliphoridae and Sarcophagidae were seen as residual elements because it is more likely that they had completed their developmental cycle at the time that the evidence was recovered. In this case, the PMI estimation was performed on the basis of developmental data of *Dermestes* species as described by Martín-Vega et al. [63] and considering the environmental temperatures of the site during the summer (about 25–27 °C as a mean), concluding a period of about 23 days from the oviposition. According to Arnaldos et al. [13], during the summer season, adults of *Dermestes* are present on a corpse from Day 7 onwards, and larvae were recorded from Day 22, reaching a maximum around Day 37. Thus, a PMI_m_ of about 30 days was estimated.
- Case 7. A male corpse was found inside his home in Jumilla (Murcia province), lying on the ground and wrapped in a blanket, on November 19.
  According to the testimonies, the man was last seen alive on November 8. Entomological evidence was collected during the removal of the body and from the autopsy procedure. In addition, a graphical report was provided. The evidence consisted of four containers, two of them with living larvae, one with adult specimens dead and dry, and the other with immature specimens in 70% ethanol. The two living samples were introduced in an incubator chamber and kept at a temperature of 25 °C and a RH of 60%. Most specimens had pupated in about 10–13 days, and the adults emerged about one month later. The emerged adults belonged to *Calliphora vicina*, *Sarcophaga argyrostoma* (Robineau-Desvoidy, 1830) and *Chrysomya albiceps*. Meanwhile, the dead and fixed samples contained *C. vicina*, *L. sericata*, *Ch. albiceps*, *S. nudiseta*, and *Polistes gallicus* (Linnaeus 1767), as well as larvae II and III of Sarcophagidae (probably *S. argyrostoma* because the emerged adults belonged to this species) and abundant unidentified Diptera eggs and young larvae. From an in-depth study of the graphical report, it could be deduced the existence of abundant evidence on the blanket wrapping the corpse, in particular on the areas where fluid loss had occurred, as well as in contact with the body surface. There was no evidence on the head (eyes, mouth, etc.). The overall evidence was dominated by Diptera species that are common during the season [13]. A period of about seven days was estimated on the basis of the development of *C. vicina* and *S. argyrostoma*, to which the most aged larvae belonged, according to data reported in [57] and [64]. The mean outdoor temperatures at those days oscillated between 11 and 16 °C. A more accurate PMI_m_ could not be estimated because no data on environmental indoor conditions were provided. Moreover, the fact of the house being closed, with windows and shutters also closed, must have made it difficult for the insects to access the corpse. Thus, it was considered reasonable to add a minimum period of three days, following the experience of Goff [65], to estimate the PMI_m_. This delay (colonization period) was considered on the basis of the circumstances of the scene (completely sealed building and the corpse wrapped) when compared with data from Goff [65], who stated a delay of two and a half days in the colonization of a corpse also wrapped but exposed at much higher mean environmental temperatures (20.5–23.8 °C) than in our case (11–16 °C). Therefore, an additional half-day was considered to estimate a minimum colonization period, as stated above. All considered, allowed us to conclude that death occurred around the day of the decedent’s disappearance.

## 3. Discussion

Basic sciences are the first step in the translation of scientific knowledge to practical application. Without basic research, scientific advances would be limited and, overall, knowledge would stagnate. This situation applies to Forensic Sciences and, in particular, Forensic Entomology. Thus, it is quite difficult to reach a valid conclusion concerning entomological evidence if no previous understanding of entomofauna composition, dynamics, or any other matter exists. These issues are particularly relevant in geographical areas displaying biodiversity hot spots that may contain a high number of species and where some of which have not been studied in detail. This is the case of the Iberian Peninsula.

In addition, and as it concerns Forensic Entomology practice, Spain, like other countries, presents additional difficulties because this discipline is not included in the forensic routine and is only considered a supplementary technique. Moreover, the collection of entomological evidence can only be done by the police officers responsible for visual inspection and medical examiners, and, unfortunately, just a few professionals are willing to spend the time and efforts that are needed to recover entomological evidence for expert studies. Finally, and with very few exceptions, neither police officers nor medical examiners have been appropriately trained for entomological purposes and lack expertise on what is needed for a Forensic Entomology expert report, as proposed by Amendt et al. [4,66].

We have presented some actual interesting forensic cases that exemplify the applicability of studying entomological evidence related to corpses regardless of the appropriate protocols that might have been followed by the police officers and medical examiners. Few forensic cases reports exist in Spain, which may be the result of a low number of researchers interested in Forensic Entomology, probably due to the difficult research conditions and the lack of specific academic programs focused on this discipline. According to Wang et al. [67], case reports are quite valuable because Forensic Entomology is constantly evolving and broadening with its application. Thus, in order to increase interest in the discipline in countries, like Spain, it would be important to make more information available on actual cases related to Forensic Entomology. Examples of forensic case reports of interest in a number of countries are presented [67,68,69].

We think that collecting environmental data in situ or worrying about obtaining it both in situ and from the meteorological station should be done, even when dealing with an indoor location. This is a recurring issue in all the cases we studied. In Case 1, the absence of entomological evidence other than a reasonable graphical report made the conclusions, by themselves, weak. Despite that, such conclusions allowed the judicial police officers to open a new line of research that was finally successful and supported our statements. It is curious that we are usually responsible for obtaining data from the weather station. This situation can be problematic, especially in cases such as Case 7, where the indoor environmental conditions were unknown and only an approximation to the PIA could be achieved.

As indicated above, another limitation of the current forensic practice dealing with Forensic Entomology concerns the lack of training in the procedures to be followed in collecting evidence at the scene or from the corpse. This issue has been highlighted in all the presented cases, where a lack of appropriate collection of evidence made it difficult to achieve expert conclusions. Some examples of nonoptimal techniques concern the collection of a nonrepresentative sample of the existing fauna on the corpse (Cases 2, 4, and 7), bad preservation of living samples that can arrive dead to the lab (Cases 6 and 7), inadequate fixing of larvae (Cases 3, 5, and 7), or the lack of entomological evidence except for a graphical report (Case 1), which was of great quality. All the above highlight the need for providing specialized training in Forensic Entomology procedures to the different agents involved in a forensic investigation if the aim is to apply entomology to such forensic investigation.

Despite all the above, an expert report could be provided in the presented cases thanks to the previous knowledge obtained from faunistic, ecological, biological, etc., studies on entomosarcosaprophagous fauna, not from our lab in Southeastern Spain but also from other researchers. Thus, when considering the fauna collected as a whole, we were able to compare it with the results of former studies on seasonal cadaveric fauna in the area, allowing us to confirm both the environmental and seasonal origin of the studied samples.

As indicated above, knowledge of the morphology of the different species allows the accurate identification of the specimens, even when no complete individuals are available but only fragments of activity remain. Such is the situation of Case 2.

Despite all the available data on the life cycle and developmental patterns of some of the main species of forensic interest, much research is needed for more species and in more geographical areas. This is an especially important issue in areas such as the Iberian Peninsula, with high biodiversity and significant endemism in certain groups. Furthermore, the Peninsula, due to its particular geographical situation, has a high potential level of exchange between populations of different ranges.

An example of a case that could be solved thanks to the different previous studies here mentioned (faunistic, morphological, and developmental) is reported in Arnaldos et al. [34]. Moreover, this case allowed reporting for the first time a species related to a corpse (*Telomerina flavipes* (Meigen, 1830)), enlarging the global list of Diptera of forensic importance. This case, in addition, emphasizes the need for a proper collection of evidence and the identification of all entomological evidence to the deepest possible level in forensic practice because the recognition of new species involved in this process could provide additional information related to PMI [34].

All the above issues have been previously discussed by other authors in different countries. Thus, not much novelty regarding new taxa or new developmental data is now presented, although it reinforces the need for performing basic studies on taxonomy, ecology, succession process, etc., that can be applied to forensic practice. As stated by Saloña-Bordas et al. [35], although major advances in Forensic Entomology have occurred in Spain, further efforts are needed in the basic aspects of fauna related to corpses in different environments and regions. New trends in Forensic Sciences related to entomology point to microbiome [70], genetics [71], and cuticular hydrocarbons [72,73] studies among others; however, if one does not know what insect species are involved the features of the succession process in a given area, all attempts to deepen in the other, and newest, aspects would be predicted to be unsuccessful. It is important to remember that the first and critical attribute to be discerned concerns the fauna related to a corpse and its state and both aspects vary according to the environments, regions, and seasons.

## 4. Conclusions

The cases presented here demonstrate the utility of performing biological studies of all the entomological fauna of forensic interest. While the general opinion may be that most of the obtained results of such studies are not directly applicable to the forensic practice, the tremendous variability of forensic scenes and situations (corpse size and condition, wounds, wrapping, type of habitat, season, particular environment, etc.) validate the fact that almost every result is useful for providing an accurate expert report when needed.

## Data Availability

Not applicable.
